# The Resonance Structure of Raman Scattering for Emitted and Absorbed Phonons in Chirality-Pure Carbon Nanotube Films

**DOI:** 10.1021/acsnano.5c12607

**Published:** 2025-11-14

**Authors:** Paul Finnie, Adam Wind, Jianying Ouyang, Pavel Shapturenka, Jeffrey A. Fagan

**Affiliations:** † Quantum and Nanotechnologies Research Centre, 6356 National Research Council Canada, 1200 Montreal Road, Ottawa, Ontario K1A 0R6, Canada; ‡ 8430 University of Waterloo, 200 University Avenue West, Waterloo, Ontario N2L 3G1, Canada; § Materials Science and Engineering Division, National Institute of Standards and Technology (NIST), Gaithersburg, Maryland 20899, United States

**Keywords:** single-wall carbon nanotubes, Raman spectroscopy, Raman excitation map, Stokes, anti-Stokes, Raman excitation profile

## Abstract

Chirality-pure single-wall carbon nanotubes (SWCNTs) are ideal samples for resonant Raman scattering (RS) as they have sharp optical transitions and are strong Raman scatterers, having diameter and chirality specific resonances in the visible and in the near-infrared. Recently, it has become possible to rapidly obtain broadband maps of many vibrational modes of SWCNTs and their excitation resonances. Here, we use a full spectrum Raman excitation mapping technique to obtain experimental Raman excitation maps for several species of SWCNT [(7,6), (7,5), and (6,5)] purified by two different methods, mapping both phonon emission (Stokes) RS and phonon absorption (anti-Stokes) RS. These experimental maps show intricate patterns which match well with some of the predictions of quantum models of RS, and strong signals enhanced ≈3000× over graphite G bands near incident photon resonance. All RS bands necessarily have different excitation structure, but Stokes and anti-Stokes pairs are closely symmetrical, yielding an overall butterfly shaped pattern interpretable in terms of excitonic resonances with incident photons and scattered photons. Temperature provides the phonon population for anti-Stokes RS and is critical to interpreting relative feature intensity compared to Stokes RS. SWCNTs are robust and strong RS, both Stokes and anti-Stokes and their ratio, can be obtained over orders of magnitude in laser power, which we show is a critical variable affecting relative response based on sample preparation factors.

## Graphical abstract

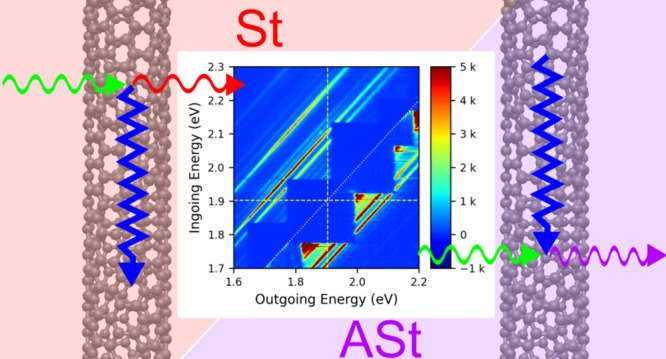

## Introduction

Raman scattering (RS) reveals vibrational modes of molecules and materials. For single-wall carbon nanotubes (SWCNTs),^1^ among many other materials, RS is an essential characterization tool. When photons - either incident or scattered - are at energies which match electronic resonances of the sample, scattering probabilities are enhanced, and the process is called resonant RS (RRS). Different molecular structures (species, or colloquially “chiralities”) of SWCNTs, which are classified by two whole number indices (*n*,*m*) identifying the “roll up” lattice vector on a graphene sheet that would form a seamless cylinder, have different vibrational modes and different electronic (/excitonic) resonances.^2^ The widely used Kataura plot,^1–4^ which maps the peak resonant wavelength (usually in the visible for the *E*_22_ resonance) versus the radial breathing (RB) vibrational mode frequency (≈150 to 350 cm^–1^), allows one to infer the diameter and even the exact (*n*,*m*) of individual SWCNTs. This mode is most commonly called the radial breathing mode (RBM), however here we use RB for brevity. (It is a peculiarity that this band alone includes “M” for “mode” in its most commonly used notation, while all the other bands do not, so RB is arguably a more consistent choice for nomenclature.) The resonance excitation profile (REP), *i.e.*, the RRS intensity versus wavelength, of single Raman modes such as the G band (≈1592 cm^–1^) has also been explored for a number of (*n*,*m*) species, revealing quantum interference effects and stimulating interest in RRS photophysics.^5–8^ More generally, SWCNTs have many RRS bands, with each having its own particular REP.

Until recently it has been onerous to obtain REPs over a broad range with high resolution due to the need for a high-quality light source, such as a tunable laser, which has limited ranges and nontrivial tuning. Progress in optoelectronics, such as the hyperspectral method used here (*vide infra*), however, are making it easier to build up multiwavelength- essentially full color - plots of RS intensities mapped out versus incident and outgoing photon energies.^9–11^ These are termed Raman excitation maps (REMs) and include both RS spectra and REPs as subsets. The Kataura plot can be viewed as the REM for the RB mode alone, and by itself is an important fingerprinting tool for SWCNTs. More broadly, every mode necessarily has a related, but different, REP, and SWCNTs have multiple vibrational modes, many of which produce strong RS. It is interesting to investigate all these modes together and observe how they change with ingoing photon energy (i.e., laser wavelength), both in terms of photophysics, because they reflect intrinsic material properties and coupling to their environment, and as a potentially very selective and sensitive characterization tool for general chemical analysis.

In Raman scattering, both phonon emission, known as Stokes (St) scattering, and phonon absorption, known as anti-Stokes (ASt) scattering are possible. Early studies on SWCNTs had to contend with mixed chirality ensembles and it was quickly apparent that fixed wavelength RS sampled different populations of SWCNTs in St and in ASt.^12^ Single SWCNTs RS studies demonstrated wavelength dependent St/ASt peak ratios for the RB mode and rationalized this in terms of the different resonance enhancement structure for St vs ASt.^13^ More recently, the G band REP has been investigated in St and ASt for chirality sorted SWCNTs, showing asymmetric REPs with different peaks for St and ASt.^14^ Beyond this, broadband REMs have not been applied to measuring St versus ASt phenomenon on the same SWCNT sample; it is interesting to look at both St and ASt together, to see how they compare, and experimentally evaluate what more can be learned from the combination.

If they are reasonably easy to obtain, there is great potential for more specific and sensitive chemical sensing using REMs. There is also great potential to test photophysical models of materials and light scattering. As an analytical tool the REM is exquisitely specific, containing all RS information, all REP information and the coupling between them. Just as the RB mode Kataura plot “unfolds” the RB region and the photoluminescence excitation (PLE) map unclutters fluorescence spectra, all RS can be sectioned in this way – and from scattering theory, every band behaves differently and has its own profile. REM also has potential to separate out physical phenomena arising from density of states, temperature and other effects. Each RS band has its own coupling to electronic states and their variation in magnitude is manifest in the REM. It should become possible to rigorously test detailed models of RS since they make different predictions for REPs and REMs. If REMs are slow to acquire, such investigations become time-consuming and less practical.

In this work we show experimental broadband REMs for species-pure SWCNTs. We show how all RS modes are affected by resonance effects on both sides of the laser Rayleigh (Ry, elastic scattering) line. Each REM is information dense, reflecting details of material properties and quantum mechanical interactions, and so can be used as a specific, sensitive fingerprint - more specific than RS alone or optical absorption alone would be – or as a vehicle to explore detailed photophysics. The St/ASt map is also sensitive to the electronic density-of-state occupancy, and hence the temperature.

Most commonly, RS is performed with fixed wavelength laser illumination. This laser light is typically focused to a spot onto a sample by a microscope objective, and the scattered light is collected, often by the same objective, with the elastically scattered laser light blocked by filters and the remaining scatter dispersed by a grating for spectral analysis.^15^ Since SWCNT show highly resonant RS, often two or more laser wavelengths are used in sequence to characterize samples. Taking advantage of progress in optical equipment, we have been using a generalization of this method that we call full spectrum REM (FS-REM).^9–11^ We use a broadband white light source dispersed spectrally across the sample and focused laterally, making a kind of “rainbow” line.^16,9–11^ This light is collected simultaneously from each spatial (i.e., ingoing photon energy) position, filtered, and dispersed spectrally across a 2D detector. This makes it possible to simultaneously collect the RS for a broad wavelength range of ingoing photon energies in a single capture sequence, such that we can collect the entire REM very quickly with acquisition times in the range from milliseconds (in the case of single capture windows where we do not change any of the Ry line blocking filters) to minutes (across the full range of ingoing photon energies and outgoing photon energies in the case of a weaker scatterer).^11^ Though ASt scatter is much weaker than St scatter, the signal-to-noise on our FS-REM instrument is adequate to readily obtain experimental ASt REMs from SWCNTs with exposure times as short as seconds. For a fixed spectral window, here we acquire St and ASt in separate exposures using different filters, and to cover a large bandwidth we combine several smaller spectral windows using several different filter pairs. This approach to blocking unwanted Ry light scattering and passing wanted RS is extremely efficient, however, note that while the resulting stepwise maps cover large Raman shifts well, lower energy bands such as the RB mode end up getting blocked intermittently. More experimental details are in the Methods section.

At the same time as there has been progress in optical instrumentation, there has been progress in separation science, such that highly (*n*,*m*) pure SWCNT samples are available in practically useful quantities for experiment and application.^17,18^ Here, we investigate three different separated (*n*,*m*) species of semiconducting SWNCTs, prepared in different ways, and report experimental broadband St/ASt REMs for each of them. A detailed description of sample preparation is in the Methods section. Briefly, all three species, (7,6), (7,5) and (6,5) were prepared by aqueous two-phase extraction (ATPE). Samples of the (7,5) and (6,5) species were also separately prepared by a polymer wrapping procedure, which, for the polymers used here, often goes by a more specific technical name: conjugated polymer extraction (CPE). This acronym is used below. To distinguish the two sets of samples, we append an “s” after the (*n,m*) indices to those prepared by ATPE, and a “p” to those prepared by CPE.

Homogenous, flat opaque or semiopaque films are ideal for rapid REM measurements and several deposition methods were used. Liquid samples can also be measured this way, but unless the liquid is opaque the focal depth tends to be larger, which can cause some loss of spectral resolution, since the rainbow line is the only “slit”. Also, for liquids, light may scatter from the container, which can require additional care. Opaque films scatter light very effectively, with no container required. Scattering intensities are concentration dependent, so liquid samples, if they are dilute, tend to have correspondingly lower intensities. Of course, just like conventional RS, inhomogeneous films can be scanned and/or averaged, but homogeneity is very convenient to enable “one-shot” measurements that are fully representative of the sample under investigation.

The ATPE samples were all dropcast and dried on CaF substrates, except for one of the (7,6) samples, denoted “(7,6)­s,Si”, which was produced by membrane filtration and transferred as a semiopaque thin film onto a silicon substrate removing the surfactant during the process. CPE samples were deposited by filtration into a film on a polytetrafluoroethylene (PTFE) filter membrane. Except for the “Si” appended sample, it is worth remembering that “s” samples remain in a surfactant matrix, and “p” samples coated by their conjugated polymer.

## Results and Discussion

Figure 1 shows an REM for the specific sample labeled (7,6)­s,Si, which signifies the (7,6) species prepared in surfactant and deposited on Si. The ingoing photon energy, from the supercontinuum laser, is along the *y*-axis ranging from 1.55 eV (800 nm) to 2.55 eV (486 nm). The detected outgoing photon energy, along the *x*-axis, ranges from 1.45 eV (855 nm) to 2.45 eV (506 nm). The intensity, in counts/s, at each pixel on the camera is represented by the color scale. The elastic (Rayleigh) scattering line, along which the ingoing photon and outgoing photon energies are equal, is represented by the diagonal dotted white line, labeled Ry, following terminology for elastic light scattering by molecules. Here, the multiple blue step-like overlapping squares adjacent to the Ry line are instrumental and caused by the filters used to block the elastically scattered laser light. RS, by definition inelastic, is observed as diagonal lines of the same slope as the Ry line (slight deviations are possible), but of varying intensity by band, (i.e., the intensity of the particular phonon mode) and along any given band (i.e., the intensity variation along the length for a given phonon mode) due to interactions with electronic states.

**Figure 1:**
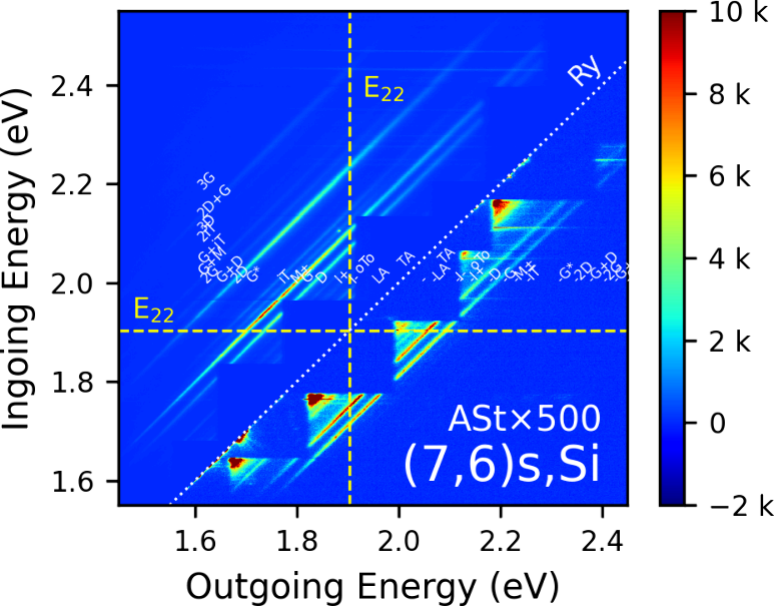
Experimental Stokes/anti-Stokes Raman excitation map shown for a surfactant process prepared (7,6) on silicon, labeled (7,6)­s,Si. The laser energy (eV, i.e., proportional to the inverse of the wavelength) is on the *y*-axis, the scattered light energy (eV) is on the *x*-axis. The color scale is the intensity in counts/sec at each pixel on the camera. Raman bands are diagonal lines of slope equal to one. These Raman bands are labeled in white letters with their common names taken from tables (see text). The dotted diagonal line is the elastic scattering line, labeled Ry for “Rayleigh”. Stokes (St) scattering is above-left of this line. Anti-Stokes (ASt) scattering is bottom-right of this line, and is scaled by a multiplication factor of 500, (labeled ASt×500) to have a similar intensity to the St scatter. The experimental *E*_22_ excitonic resonance is labeled on both axes by the dashed yellow line.

Conventional St RS, in which phonons are emitted in the scattering process, are above and to the left of the Rayleigh line (lower outgoing photon energy than ingoing photon energy). ASt RS, which involves phonon absorption, is below and to the right of the Ry line (greater outgoing photon energy than ingoing photon energy). ASt RS is typically much weaker than conventional St RS. Here, to plot both on the same scale, the ASt region has been multiplied by a constant factor to bring the ASt G band up to the same order of magnitude in intensity as the G band in the St region. This factor is displayed on the map as the number following “ASt×”.

In Figure 1, the map is additionally labeled with the second-order optical transition (*E*_22_) exciton energy for the (7,6) SWCNT on both axes. The energy of this resonance was determined by optical absorption (OA) of the purified nanotube liquid suspensions before substrate deposition. The diagonal bands are autolabeled with conventional Raman scattering phonon mode names using names from tables^19^ (TA, LA, I^–^ [short for IFM^–^], oTo, I^+^ [short for IFM^+^], D, G, M [combined M^+^/M^–^], iT [short for iTOLA], 2D [also sometimes called G’], G+D, and 2G). Each St mode has a corresponding ASt mode of the same label but with a minus sign in front. In St, some higher order modes beyond those typically tabulated are also visible and can be assigned as described in earlier contributions (e.g., G+M, G+iT, 2iT, 3D, 2D+G, 3G).^9–11^

Notably, the variation of the incident laser power with ingoing photon energy, and the variation of the response of the optical system is not corrected for in Figure 1 or maps in subsequent figures. However, it will be shown below that overall, for this instrument, they could be corrected with a linearly scaling correction factor over the ingoing photon energy range from 1.6 to 2.35 eV. Above this energy the correction is nonlinear because the intensity of the light source drops. We do not apply this correction to the maps, but will apply it to REPs, below.

Figure 2 shows the same type of REM plot, but for multiple species, including the (7,6), (7,5) and (6,5), and several different sample preparations. Figure 2a, is the same (7,6) map as Figure 1, shown again here for comparison purposes. Figure 2b,c are (7,5) and (6,5) SWCNT films respectively, both on PTFE substrates, and having been prepared by CPE. Figure 2d–f are the same sequence of species as the top row, (7,6), (7,5) and (6,5) respectively, but sorted by ATPE and dropcast on CaF substrates. Having different species and different preparations enables the evaluation of systematic changes.

**Figure 2:**
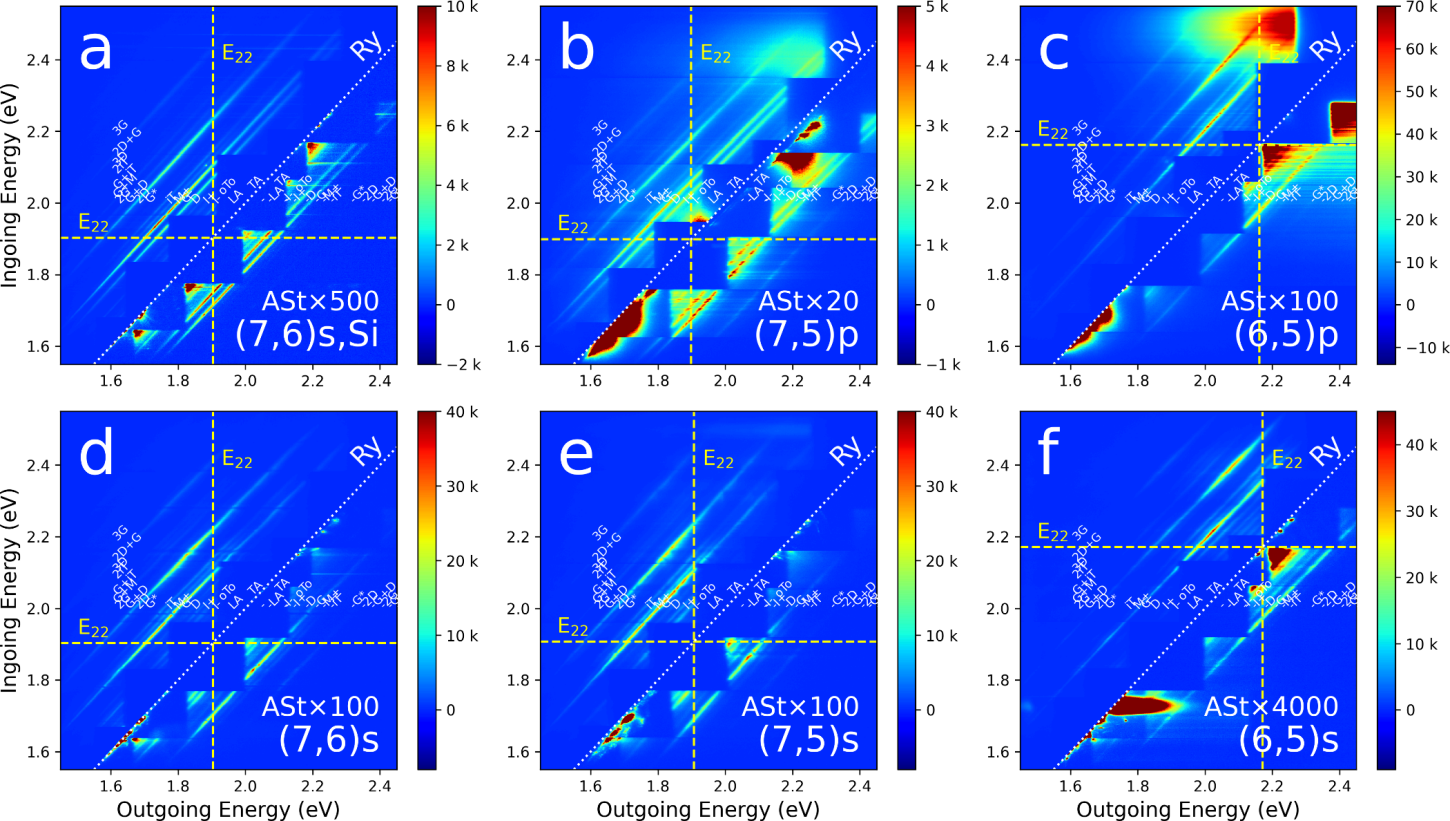
Experimental St/ASt REMs as measured for three different SWCNT (*n*,*m*) samples prepared in different manners. Top row: (a) surfactant prepared (7,6) on silicon (b) polymer wrapped (7,5) on a PTFE membrane (c) polymer wrapped (6,5) on PTFE. Bottom row (d–f): the same (*n,m*) species as shown in the top row, but deposited by drop casting from surfactant dispersion on CaF substrates. Labeling of spectral features is as in Figure 1. Similar RS features are observed across all REMs, both in St RS and ASt RS, however with significant differences in the resonance energies, comparing across (*n,m*)­s, and in the intensity of the observed St *versus* ASt RS as a function of increasing distance from the diagonal, elastic, Ry line scattering feature. Note that the ASt RS has been multiplied by a different value in each panel, labeled as “ASt × ” such that the ASt and St RS are comparable with the same color intensity scale.

The REMs in Figures 1 and 2 are rich with detail and we highlight some of the major features. Among the most intense bands are the D, G, 2D and the ASt -G band, so we emphasize them. Examining the maps, in all cases their intensities are highest in the top-left and bottom-right quadrants of each panel as delineated by the *E*_22_ resonance energy, shown as horizontal (ingoing photon energy) and vertical (outgoing photon energy) dashed lines. Intensities of the stronger RS bands are of the order of 10k counts/s in the top left St quadrant between the horizontal and vertical *E*_22_ lines, and similarly intense in the bottom right ASt quadrant between *E*_22_ lines, but only after multiplying by the ASt factor shown on each plot. Crossing these dotted lines, i.e., moving outside these quadrants, the band intensities drop significantly, though they only weaken and are still detectable. In the case of Figure 2b they only weaken by a limited amount moving up and to the right.

For comparison, using a uniform intensity scale, many more RS bands are visible on the St side than on the ASt side. On the ASt side, the −2D is present on most ASt plots, but barely visible on the scale of Figure 2. Supporting Information Figure S1 shows the same data on a color scale which brings out weaker features while saturating stronger ones. Then the weaker −2D band can be seen faintly in (a) and (c) and more strongly in (b), (d) and (e). The intensity of ASt bands drops off moving down and to the right, away from the Ry line, in a way that St lines do not.

A clear trend emerges if we follow any one St/ASt pair of bands moving from bottom left to top right. With the choices of color scale this is easiest to do for the G/–G bands. It is apparent that the intensities of the two bands track each other closely (as reflected across the Ry line) moving up and to the right. In fact, to a good approximation, the trend of the ASt band is the mirror image of the St band across the diagonal Ry line. Below, this trend will be rationalized with reference to the most basic quantum mechanical pictures of RRS.

There are, however, some other complications to keep in mind. Dropcast samples (Figure 2d–f) are not as uniform as filtered or filtered and transferred samples (although having other advantages), and this spatial nonuniformity can cause some variation in these single-shot REMs. On all maps there are occasionally bright horizontal streaks which are artifacts from extra light scattering due to particulates on the surface. Less apparent, there are also dark horizontal streaks arising from places where the scattered light is either blocked by particulates, or possibly where there are no nanotubes. The CPE samples, in addition to St and ASt Raman, also show broad light emission at high laser energies which is attributable to fluorescence from the polymer surrounding the SWCNTs.^11^ Importantly, the multiplication factor required to bring the ASt intensity up to the St intensity in the shown REMs varies widely, from as low as 20 in Figure 2b to as high as 4000 in Figure 2f. This will be explained below.

Other features in Figure 2 are physically interesting. In line with previous studies,^5–8^ for the G band the lower ingoing photon energy resonance (where the ingoing photon is at energies close to *E*_22_) is consistently stronger than the higher energy resonance, except for the (7,5) sample for which this is not clear. However, not all bands are like that. The 2D band is greater near the outgoing photon energy resonance, with the peak down shifted slightly from the actual *E*_22_ line. The efficiency of the optical system in ingoing photon energy and outgoing photon energy will bias the signal somewhat, as will be seen below, but it does not account for this large difference, especially in comparison to the other samples.

Next, we interpret some key aspects of the maps in the context of the quantum theory of RS. Although the theory of RS is very well developed, there are several descriptions corresponding to various levels of approximation in the scattering process, using different orders of perturbation theory,^5,6,20,21^ assumptions about whether real or virtual states are involved,^20,21^ and assumptions about the motion of atomic nuclei.^5,6,21^ The various models may predict similar single laser wavelength RS, but should make quantitatively different predictions for REPs and REMs. Hopefully, with REM becoming more accessible, comparing experimental maps with detailed models could help confirm what picture best represents the real physical process and the importance of specific terms.

Without specifying any particular model, there is an interesting fundamental feature of the St/ASt REMs. That is, St RS is the time reverse process of ASt RS. The scattering probability is symmetric under time reversal, which on the map amounts to swapping ingoing photon and outgoing photon energies – i.e., reflecting the map across the diagonal Ry line. In fact, this symmetry is already apparent by eye to a good approximation if you look at any one band, e.g., if you compare the shape of G and -G bands for any of the maps. However, all ASt bands are attenuated from St bands and they are attenuated by different ratios, so this symmetry is broken when looking at more than one band.

Briefly ignoring the attenuation, there are different models used to calculate the RRS probability. One model is second order Kramers–Heisenberg–Dirac (KHD) theory,^5,21^ which can represent SWCNT REPs well if four levels are used.^5^ Another popular treatment of the quantum theory of RRS is third-order perturbation theory, described in ref 22, and also presented in a nanocarbon specific context in ref 19. We follow the latter here.

The basics of this treatment follow from considering the three involved particles: the incident photon, the scattered photon, and the phonon that is emitted in St RS (or absorbed in ASt RS). All three interact with the system of electrons which gives rise to six permutations, which become additive terms for the scattering matrix element, all of which both add coherently and interfere.^22,19^ This is simplified mathematically by considering only the most highly resonant terms, that is, those that have denominators tending to zero as the ingoing photon approaches some energy. In the limit when the phonon energy (*Q*) is small, one of these terms has a twin resonance enhancement and the matrix element can be simplified to

$$P\propto {\left|\frac{H}{\left(E_{L}-E_{X}-i\Gamma )(E_{L}-Q-E_{X}-i\Gamma \right)}+C\right|}^{2}$$ 1

Here *H* is an effective scattering matrix element integrated over many variables, *E*_L_ is the laser energy (i.e., ingoing photon energy), *E*_X_ is the exciton energy, *Q* is the phonon energy, Γ is a damping term which softens singularities, and *C* is a sum of the five other scattering terms which are all presumed small because they do not have the twin resonance. Eq 1 is for St but is the same for ASt, only substituting −*Q* for *Q.* (*Q* is also present in the *C* term.) In general, light scattering, whether Rayleigh, St RS or ASt RS also has a nonresonant scaling factor multiplier for all terms which is nearly fourth power in *E*_L,_ but this factor is not explicitly written out in eq 1.

The first term has two potential zeros in the denominator: one from the incident photon when *E*_L_ = *E*_X_ and one from the scattered photon, *E*_L_ = *E*_X_ + *Q* (for ASt RS these are at *E*_L_ = *E*_X_ – *Q* and *E*_L_ = *E*_X_). In the *C* term, none of the additive terms has the twin resonance, however there are terms which are singly resonant at *E*_L_ – *E*_X_ and also *E*_L_ – *Q* – *E*_X_ (*E*_L_ + *Q* – *E*_X_ and *E*_L_ – *E*_X_ for ASt). If the phonon is low enough in energy, these two energies of vanishing difference overlap, and the twin resonance makes the first term dominate over *C*. This is a good approximation when the phonon energies are small, e.g., likely for the RB mode (≈20 meV). This term’s dominance becomes weaker as *Q* increases in energy, because it is not possible for both incident and outgoing photons to be resonant. In that case, there is only one zero in the denominator and the singly resonant terms in *C* have the potential to become non-negligible. As one factor in the denominator of the leading term approaches zero, the other tends to *Q* (0.2 eV for the G band). Due to the singly resonant terms hidden in *C*, unless they happen to coincide exactly, this already makes the scattering probability asymmetric with respect to swapping *E*_X_ with *E*_X_ + *Q* (or *E*_X_ with *E*_X_ – *Q* for ASt).

If the *C* term is neglected (i.e., set to zero) and the *H* element considered constant – both probably mathematical conveniences more than physical realities – the above simplifies to

$$P\propto {\left|\frac{1}{E_{L}-E_{X}-i\Gamma }-\frac{1}{E_{L}-Q-E_{X}-i\Gamma }\right|}^{2}$$ 2

This makes for a double peak structure. However, this simplified equation indicates an equally strong resonance at *E*_X_ (incident) as at *E*_X_+ *Q* (scattered) for St RS, which is not observed experimentally (*E*_X_ – *Q* and *E*_X_ for Anti-Stokes RS). The nearly fourth power factor in *E*_L_ for nonresonant scattering is not written explicitly here, just the resonant factor. Asymmetry was explained in terms of the second-order model originally, where it was interpreted as a consequence of nuclear motion and the breakdown of the Franck–Condon principle, the approximation that electronic transitions are instantaneous compared to any nuclear motion. However, asymmetry also comes out of the third-order theory when instead of considering a single excitonic level, a sum is taken over a more realistic model of the electronic density of states. For simplicity, drawing from the original treatment, the asymmetry can be parametrized by a constant, ε, such that a reasonable expression could be

$$P\propto {\left|\frac{1+\varepsilon }{E_{L}-E_{X}-i\Gamma }-\frac{1-\varepsilon }{E_{L}-Q-E_{X}-i\Gamma }\right|}^{2}$$ 3

(again leaving the nearly *E*_L_^4^ factor implicit). A schematic of the general structure of the REM that arises just by considering these two resonances, and considering only one electronic (here more specifically, excitonic) resonance is shown in Figure 3a. Suppose there is one single electronic resonance, *E*_X,_ and one single phonon energy *q*. Then, there will be a St RS line parallel with *q* above the Ry line, and an ASt RS line parallel with *q* below the Ry line. If the laser is at energy *E*_X_, both St and ASt will be resonant, but as the laser energy is increased, the ASt will go off resonance while the St will remain on resonance until the laser reaches *E*_X_ + *q*, at which point it will start dropping. Decreasing the laser energy from *E*_*X*_ will do the opposite. The intensity should be peaked near *E*_X_ and *E*_X_ – *q* for St, and *E*_X_ and *E*_X_ + *q* for ASt, and may show a complex evolution in between due to quantum interferences. Here it is illustrated as dropping off to zero suddenly at both the ends – in reality, it will decay and could show interference effects there as well.

**Figure 3:**
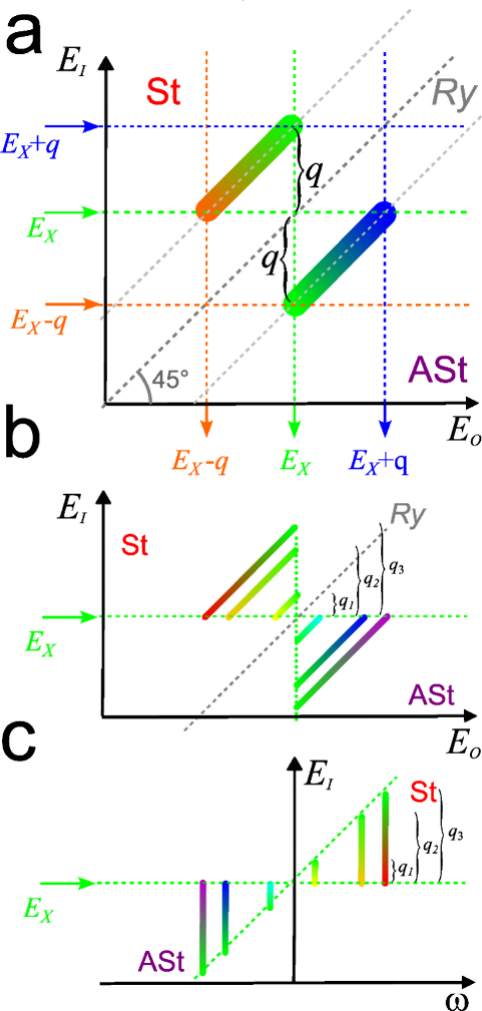
Simple schematics of expected structure of the ingoing photon energy-outgoing photon energy map. (a) Schematic of a single phonon mode of energy *q* and its resonance with a single electronic (/excitonic) transition of energy *E*_X_. The *y*-axis is the ingoing photon energy, *E*_I_ (*i.e.*, set by the laser wavelength) the *x*-axis is the outgoing photon energy *E*_O_ (*i.e.*, set by the scattered light wavelength). The dotted gray diagonal line is the elastic scattering line, labeled Ry for “Rayleigh”. Above this line is Stokes scattering, labeled with the St, below this line is anti-Stokes, labeled ASt. The color of the bands here is artistic only, meant to represent the wavelength of the scattered photon. In the other (experimental) figures the color represents intensity. (b) Schematic for three vibrational modes of energies *q*_1_, *q*_2_, *q*_3_ for a single electronic (/excitonic) transition *E*_x_. (c) Same as (b) but transformed so that elastic scattering is at zero on the *x*-axis, so the Stokes Raman shift is increasing to the right. This may be a more familiar representation of the map.

Adding to the complexity of observed RS, many SWCNT phonon modes are actually composed of combinations or multiples of other phonons. To account formally for these would require a yet higher order treatment, but they can be understood in analogy. Multiphonon modes generate more additive terms, and potentially result in additional intermediate resonances due to their individual phonon components.

Figure 3b shows a more generalized schematic REM still considering only one electronic resonance, *E*_X,_ but illustrating three different phonon modes *q*_1_, *q*_2_ and *q*_3_. At the incident resonance, all the phonon modes, St and ASt, are on resonance. The double-peaked pattern for each individual band and the mirror symmetry about the diagonal Ry line makes a kind of butterfly shape for the REM. Rather than treating incident and scattered photon energies symmetrically, another option, which may be more familiar, is to plot the Raman shift as the *x*-axis and incident photon energy as the *y*-axis. Figure 3c shows Figure 3b transformed in this manner. Here, Raman shift is nearly constant for each mode, so in these coordinates each vibrational mode is a vertical segment.

These butterfly shapes suggest one possible explanation for phenomena observed in time-resolved surface enhanced Raman scattering (SERS) of single molecules – namely that sometimes subsets of RS modes are resonant, sometimes only St modes, sometimes only ASt modes, and sometimes the entire spectrum is resonant.^23,24^ In fixed wavelength RS, from Figure 3b,c it is clear that which modes are enhanced depends on the energy at which the laser makes a horizontal cut of the map (*i.e.*, depends on laser wavelength). So, this model naturally explains why different modes can be enhanced differently.

With Figure 3 as a starting point, more observed features of the observed REM of Figures 1 and 2 can be rationalized. The observed band intensity is typically higher in the top-left (St) and bottom-right (ASt) quadrants defined by the *E*_22_ resonance. Also, the RS intensity is often higher close to the *E*_22_ line, as predicted. A difference between the simple model and the real world is that the SWCNT *E*_22_ exciton resonance is a 1D band, i.e., it has a breadth that a single energy level would not. Also, SWCNTs do not just have *E*_22_ resonances, but have many other optical resonances.^25^ In general, the *E*_22_ resonance, and the *E*_11_ resonance, which is commonly far lower in energy^2^ than *E*_22_, are much stronger than these other, lesser resonances.

Access to St/ASt REMs create interesting possibilities for chemical and physical analysis. St/ASt can probe the same phonon but in emission (St) or absorption (ASt). The ASt band intensity compared to the St band intensity provides information about the physical phonon population. The different resonance conditions above show that St/ASt creates the possibility to study the same phonon at very different photon energies. ASt can be used to see a phonon mode that would be outside the detection range for St, and vice versa. Also, from the butterfly diagrams, St/ASt REMs provide a way to locate the electronic/excitonic energy resonance. Above we have used a simple picture with just a single ground state and single excited state. In reality SWCNTs have many electronic/excitonic states. St/ASt maps create the opportunity to access phonon distributions as well as electronic/excitonic density-of-states. Ultimately, it should become possible to use REMs to separate out contributions to the structure which come from the electronic/excitonic density of states, and contributions which come from quantum interferences between resonances such as the ingoing photon resonance and the outgoing photon resonance.

A next step, returning to a phenomenon we set aside above, is to understand the asymmetry in attenuation of St band intensity with greater phonon energy versus that of the same ASt bands. Actually, the fact that the ASt intensity is much weaker than St intensity of the same band for nonresonant Raman is well understood. In the case of St RS, a phonon is created, but in ASt RS, a phonon is absorbed – so it must be present in the first place. The population of existing phonons is activated thermally (although there is the potential for nonthermal effects^26^). To a good approximation this makes the ratio of ASt intensity (*I*_ASt_) to St intensity (*I*_St_) for any given band

$$\frac{I_{\mathrm{ASt}}}{I_{\mathrm{St}}}=(E_{L}+q{)}^{3}/(E_{L}-q{)}^{3}e^{-q/kT}$$ 4

where *E*_L_ is the laser energy, *q* is the phonon energy, *k* is the Boltzmann constant, and *T* is the temperature in Kelvin, with the intensity in terms of counts (photoelectrons), as opposed to power (Watts).^27^ For visible lasers at room temperature and phonons in the fingerprint region (100 to 2000 cm^–1^) the exponential term dominates the dependence. Thus, as the phonon energy increases, the ASt intensity decreases exponentially. This is why ASt bands closer to the diagonal Ry line are strongest in the map and lines get weaker moving to larger shifts.

This effect causes large decreases in intensity ratios for ASt peaks and has consequences when comparing peak intensities. Of importance for nanocarbons, the D/G band intensity ratio is an indicator of crystallinity. The D/G ratio changes due to resonance and is enormously different when comparing St to ASt. High crystallinity SWCNTs typically have a very low D/G band intensity ratio in St, but the D band intensity is exponentially enhanced relative to the G band in ASt because it is closer to the elastic Ry line. As a result, the St and ASt D/G ratios are not the same and should not be used interchangeably.

Another implication of eq 4 is that the St/ASt ratio can be used to measure the temperature of the sample being probed; however, if RS is resonant (RRS), an intensity ratio from a single St/ASt pair can be misleading because the resonance effect can enhance one more than another. Empirically, the St/ASt intensity ratio also varied significantly from sample to sample within the set shown in Figure 2, which can be seen by the necessary ASt multiplication factors. The reason for this variation was not immediately clear, so we also performed conventional fixed wavelength RS using a 632.8 nm laser for each of the samples. This is reported in Figure 4 for each sample, with the St portion is in black and the ASt in red. Both show peaks from the same RS bands, but with the ASt intensity getting progressively weaker as the phonon mode increases in energy. The ASt G band at ≈1592 cm^–1^ is still detectable, but is 3 to 4 orders of magnitude below the St intensity. The dotted diagonal line on each graph shows the expected ASt intensity for a St peak at the intensity of the dashed horizontal line, assuming a sample at room temperature, based on the thermal occupancy assumed in eq 4. In rough terms, the ASt intensities in Figure 4 follow the room temperature trendline quite well. However, in the REMs of Figure 2, the ASt intensity does fall off, but much more slowly. This is an indication that for the maps of Figure 2 the sample temperature for some panels is much higher than for the fixed wavelength Raman in Figure 4.

**Figure 4:**
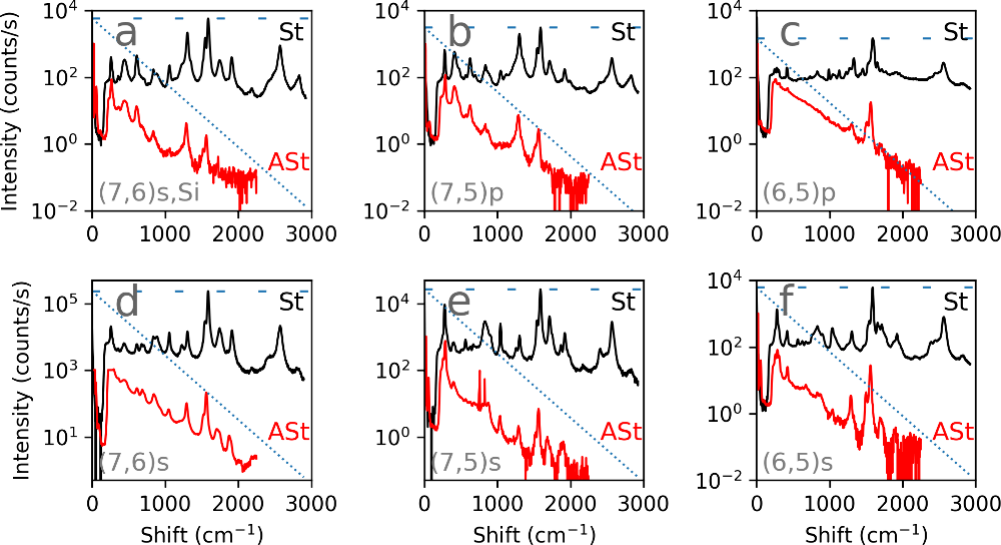
Comparison of conventional single-wavelength St and ASt RS as measured using a fixed wavelength laser (632.8 nm) for the same samples in Figure 2 arranged in the same panel order, and same letter index. St spectra are shown in black and labeled St; ASt spectra are shown in red and labeled ASt. Both are plotted on the same axis to enable one-to-one comparisons for the same RS phonon modes. Note that the intensity is shown on logarithmic scale. The dotted diagonal line represents the expected ASt RS intensity for the observed St RS from the strongest G band (dashed horizontal blue line).

Figure 5 shows two graphical views of the ASt/St intensity ratio expected from the equation for various phonon energies, some prominent band energies (labeled RB, D, G, 2D), and various temperatures. Note that rather than the St/ASt ratio, the inverse, the ASt/St ratio is plotted to emphasize the weakness of the ASt signal at low temperatures. As the temperature increases the ASt/St ratio line decreases in slope. For the G band, a ratio of 3 to 4 orders of magnitude is expected at room temperature, dropping to less than 2 orders of magnitude at 300 °C.

**Figure 5:**
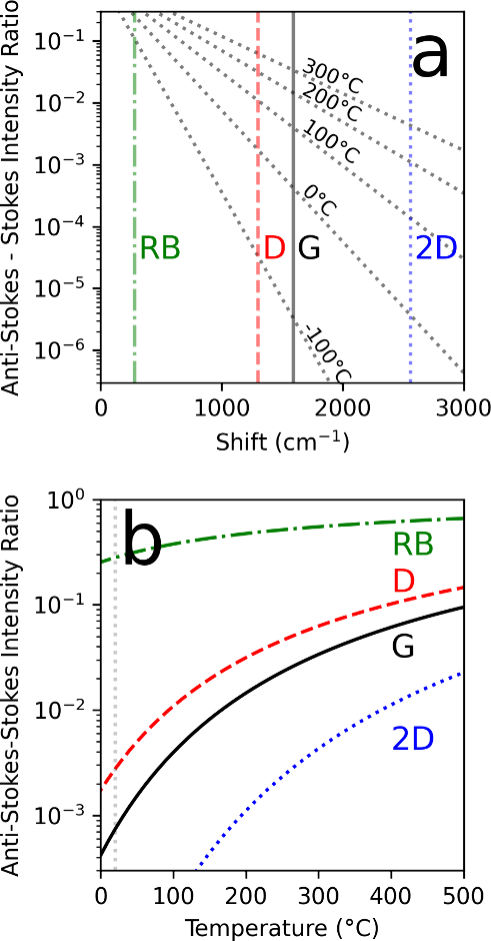
Calculated ASt/St RS intensity ratio dependencies as a function of Raman shift and temperature assuming thermal phonon populations. Two views of the expected nonresonant Raman intensity ratio in terms of counts at a given shift and a given temperature, based on eq 4 in the text. Note that the ASt/St ratio is plotted, which is the inverse of the St/As ratio. This emphasizes the weakness of the ASt at low temperature (a) ASt/St intensity ratio (unitless) as a function of Raman shift for various temperatures. The positions of the RB, D, G and 2D bands are labeled. ASt/St intensity ratio (unitless) as a function of temperature for Raman shifts corresponding to the RB, D, G and 2D bands.

With this in mind, it is understandable why the ASt intensity is different for different samples in Figure 2. They are being heated to different temperatures by the light source. The reason why the temperature is so different is mainly attributable to the timing of the sources. The HeNe laser of Figure 4 is continuous wave, meaning it is always on. However, our light source for the hyperspectral FS-REM instrument is a supercontinuum laser that is quasi-continuous, pulsing with an 80 MHz repetition rate (i.e., ≈10 ns intervals), and having a pulse duration estimated around ≈20 ps. The duty cycle is therefore 1:500, and the instantaneous power is thus 500× the average power. Also, the microscope objectives here are twice the magnification (20×) of our earlier work,^9–11^ suggesting a 4 times higher power density. Plainly, we are reaching a regime in which a lot of heat is photogenerated. The amount of heating is sample and substrate dependent, leading to the variation in ASt multiplication factor required.

To confirm that the St/ASt ratio was being affected by the power density, we changed the power density over 3 orders of magnitude using neutral density filters in the ingoing beam path, while examining sections of the REM in both St and ASt for the (7,5)p sample (Figure 6). Actually, without any neutral density filter in the beam path (100% transmission), samples were damaged too quickly in air to obtain usable ASt maps. For neutral density (ND) filters ND10, ND20 and ND30 (nominally 10, 1, and 0.1% optical transmission across all wavelengths) we were able to measure both St and ASt RS without any significant degradation of the sample on time scales of the measurement.^11^ Figure 6 shows the St (top row) and ASt (bottom row, shaded) for each of these filters, decreasing in laser power from left to right. The integration time is increased as the optical transmission is decreased so that the total photon dose is the same moving left to right. The top, St row varies little, being a fraction weaker for the ND30 filter, which is probably just that that the filter is slightly less than 0.1% transmission. On the other hand, the ASt intensity varies widely (bottom row – note color scale changes). ASt was always weaker than St, as expected (the ASt integration times here were 10× longer than the corresponding St integration times). As the power changes, the color scale on the bottom ASt row is changed by orders of magnitude. The G band intensity ratio (unitless) goes from ≈10 with the ND10 filter, to ≈200 with the ND20 filter, and to about ≈1000 with the ND30 filter. So, from Figure 5 we estimate that heating is basically negligible with the ND30 filter, about 100 °C with the ND20 filter, and may even exceed 300 °C with the ND10 filter. Heating was less important for the 632.8 nm laser, which was nonetheless at a high power of 1 mW incident for a continuous wave power density of order 1 mW/μm^2^.

**Figure 6:**
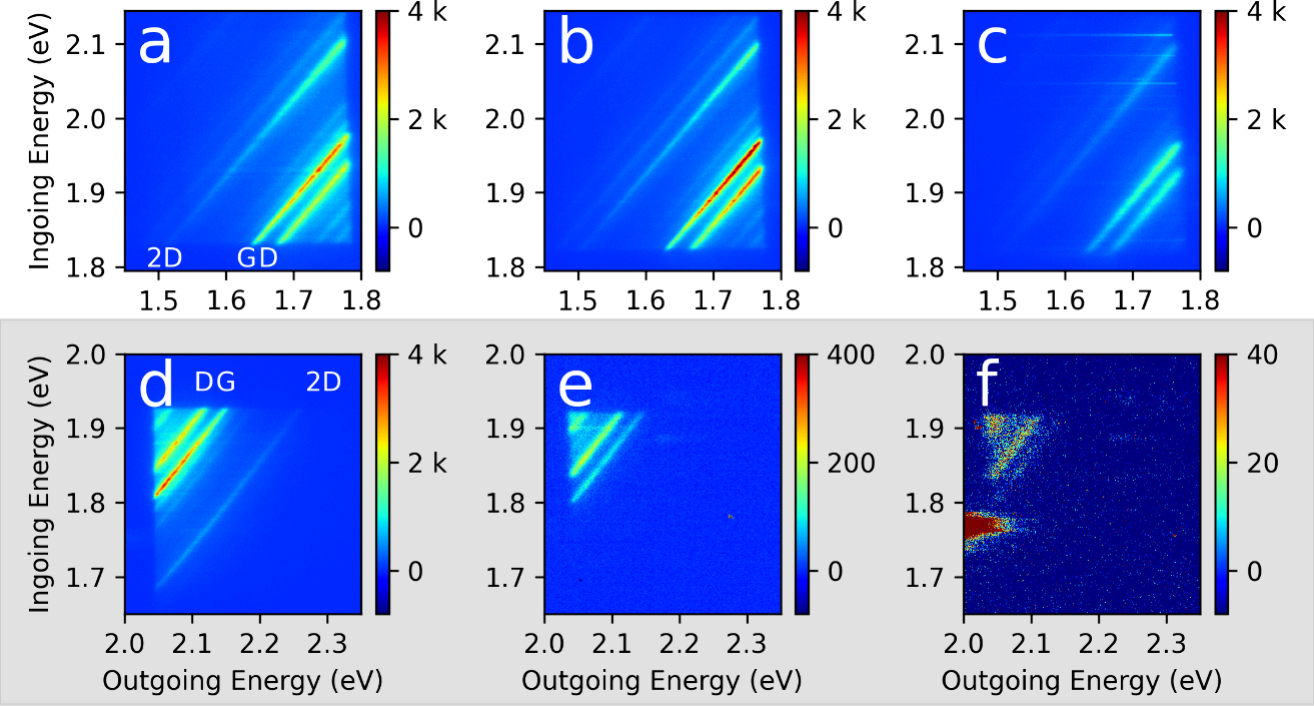
Effect of laser power on Stokes and anti-Stokes Raman excitation maps. Top row panels (a–c) show the St Raman scattering intensity for decreasing illumination power (from left to right), with the integration time scaled to hold constant the total photon dose: (a) ND10 filter (≈10% transmitted power), 1 s integration time, (b) ND20 filter (≈1% power), 10 s integration time (c) ND30 (≈0.1% power), 100 s integration time. The *y*-axis is the supercontinuum laser energy (i.e., wavelength), the *x*-axis is the energy of the emitted, scattered light. The bottom row (panels d–f), outlined in gray, present the ASt Raman scattering intensity measured at the same illumination conditions as the corresponding St REM panel, but collected for an order of magnitude longer integration time: (d) ND10 filter (≈10% transmitted power), 10 s integration time, (e) ND20 filter (≈1% power), 100 s integration time (f) ND30 (≈0.1% power), 1000 s integration time. ASt RS is much weaker than St RS in all cases, and the ratio of ASt/St RS intensities further decreases as the absolute illumination intensity is attenuated despite the panels representing collection over equal total doses of illumination. Some Raman scattering bands of carbon, the D, G, and 2D are labeled in (a) and (d). Note the 10× and 100× magnified color scales for (e) and (f) respectively. All data are for the polymer-wrapped (7,5)p sample.

From this, we can understand the variation in multiplication factor to bring the ASt map up to the St map intensities. Although the incident light intensity is the same, the alternate preparations are reaching different temperatures due to their different substrates, varying environments, and due to different optical absorptions for different concentrations and different species. Estimates of the thermal conductivities also suggest the substrate may be an important factor for heat loss. At room temperature the thermal conductivity of Si is 148 W/mK, CaF is 9 W/mK, and PTFE is 0.3 W/mK,^28^ so there could be a large difference in heat dissipation for the different substrates. We thus hypothesize that the silicon (7,6)­s,Si sample probably remains cooler than the CaF due to intimate contact with the order of magnitude more thermally conductive substrate. The polymer wrapped SWCNTs are probably heating up more due to the poor thermal conductivity of the PTFE substrates, and the polymer wrapping may also serve to thermally insulate the SWCNT. For future experiments, it will be preferable to keep the maximum power density lower and/or use less thermally insulating materials if we wish to study the intrinsic ASt REM at ambient-controlled temperatures. On the other hand, we can use photoheating to increase ASt intensities. It may also be of interest to intentionally heat SWCNTs in this way, and monitor the temperature with RS.

Finally, we consider the absolute RS scattering intensity. This provides a check on the effects of the wavelength dependent intensity of the light source, and the wavelength dependent response of the detection system. It also characterizes the true shape of the REP and the true RS resonant enhancement factor for SWCNTs. This is done by comparing to a standard, nominally nonresonant sample – here, using highly ordered pyrolytic graphite (HOPG).

Figure 7 shows the map for HOPG under exactly the same conditions as the SWCNTs in Figure 2. On the St side, G and 2D are strong and 2G and 2D+G bands are weaker, but visible. On the ASt side it is possible to discern scattering from the ASt G band, but it is very weak and buried in noise. HOPG has an in-plane thermal conductivity of ≈2000 W/mK at room temperature,^28^ so it is expected to dissipate heat well, counteracting the laser heating. The 2D and G St bands are strongest and reach intensities just above 10 counts/s. The enhancement of SWCNT RS over HOPG is already clear, since peak intensities in Figure 2 are in the range 30k counts/s for the SWCNT G band - so a factor of ≈3000× higher. To be explicit, the enhancement factor is estimated simply from the highest G band intensities in Figure 2 and the highest G band intensities in Figure 7a. We do not introduce any factor for the lower density of the carbon in SWCNTs as compared to HOPG, or for the different penetration depth, and so different geometry of the focal spot for the two materials. However, both samples are opaque, which arguably justifies this simplifying approximation.

**Figure 7:**
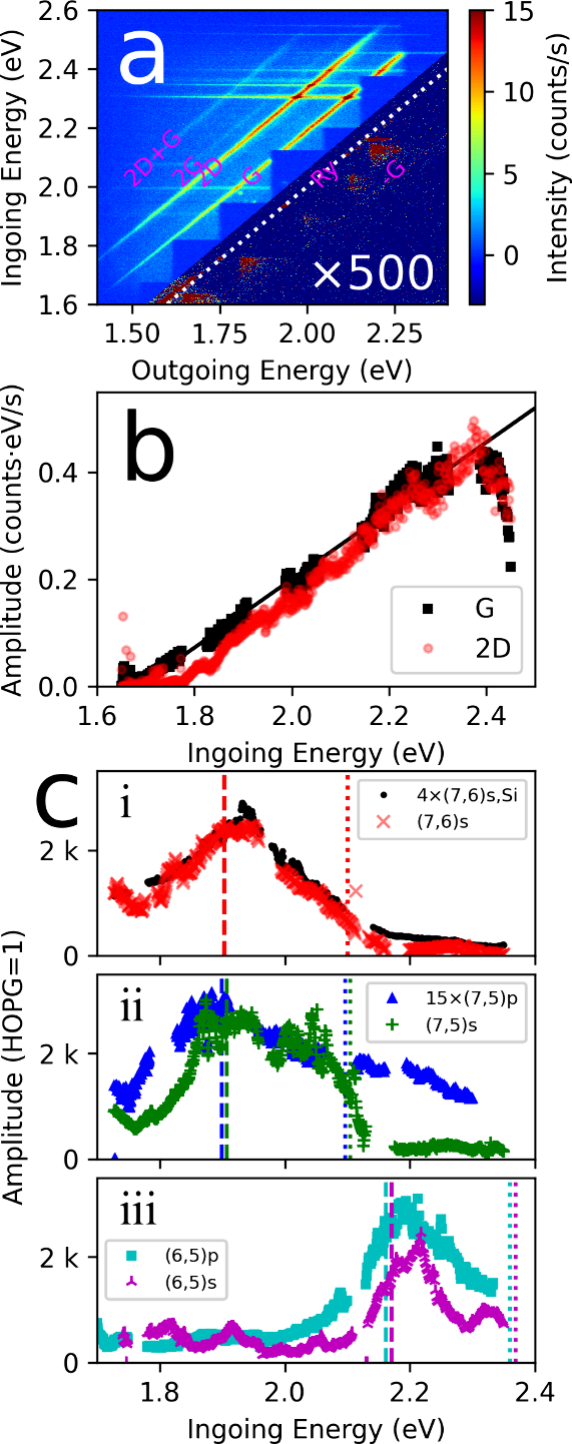
HOPG REM and HOPG corrected G band intensities. (a) Raman excitation map for graphite (HOPG) for the same experimental configuration as the SWCNT samples. The *y*-axis is the ingoing laser photon energy, the *x*-axis is the scattered light energy. The color scale is intensity in counts/s. The dotted diagonal line is the elastic scattering line, labeled “Ry” for Rayleigh. Stokes scattering is above this line, anti-Stokes scattering below. Bands are autolabeled. The G and 2D bands are prominent on the Stokes side, and the 2G and 2D+G weaker but visible. On the ASt side the -G band is barely visible even scaled up by the factor used for the Anti-Stokes intensity here, because of the large degree of noise. (b) Amplitude of the G (black squares) and 2D bands (red circles) extracted from a single Lorentzian fit. The line is a fit to the ascending part of the G band data. (c) Amplitude of the SWCNT G bands extracted from a Lorentzian fit, divided by the line fitting the HOPG G band. (i) the (7,6) samples on Si (black circles) on CaF (red crosses). The silicon sample amplitude is multiplied by a factor of 4 to match the CaF sample intensity. (ii) The polymer wrapped (7,5) sample (blue triangles) and the (7,5) surfactant sample (green crosses). The polymer sample intensity has been multiplied by a factor of 15 to better match the surfactant sample intensity. (iii) the (6,5) samples. The polymer wrapped sample is cyan squares, the surfactant sample is magenta 3-pointed crosses. For all samples *E*_22_ is marked with a vertical dashed line and *E*_22_ + G is marked by a dotted vertical line. In (i) it is the same for both samples.

To be more quantitative, the HOPG G and 2D bands were fit by simple Lorentzians and the amplitude plotted as a function of ingoing photon energy in Figure 7b. This can be used to correct for the wavelength dependence of the optical system, and also factors out the power law increase in light scattering as the energy of photons increases. The G band intensity is masked where the Lorentzian fit is distorted by the filter edges on the REMs, so it is only shown intermittently. The filter edges are far enough away that the 2D band fit covers the full energy range.

Figure 7c shows the SWCNT G band Lorentzian amplitudes extracted from the data in Figure 2, normalized to the fit line to the HOPG Lorentzian amplitude of Figure 7b. Panel 7­(ci) shows the (7,6) samples, panel 7­(cii) the (7,5) and panel 7­(ciii) the (6,5). The (7,6)­s,Si sample has been multiplied by 4× to bring it up to the same intensity as the other (7,6)­s, and the (7,5)p sample has been multiplied by 15×. The *E*_22_ excitation resonance as determined from optical absorption is labeled by dashed lines, the *E*_22_+G is labeled by the dotted lines. The polymer wrapped SWCNTs have slightly lower resonance energies than the surfactant.

At the peak of the REP, the SWCNT G band amplitude is ≈3000× the HOPG G band amplitude. The (7,5)p alone was substantially lower. It also had a much broader REP than all the others. Possibly this is because of the very high temperature this sample reached, as can be inferred from the low ASt multiplication factor in Figure 2. Also, both polymer-wrapped SWCNTs show broader REPs than the corresponding surfactant wrapped samples. This broadening may be a consequence of the high temperature.

Over the range we can see here, all G band REPs are stronger at the low-energy incident *E*_22_ resonance, and weaker at the higher energy scattered *E*_22_+G resonance. This is consistent with prior work.^5–8^ However, going back to the maps of Figure 2, it appears this does not generalize to all bands. While some do show this low-energy weighted double peak shape, such as the D band in Figure 2a, the 2D band shows the opposite behavior in all maps, peaking near the scattered *E*_22_ resonance in all case. This is the case even for the (6,5) where this trend could not be explained by the weakening detection leg response at lower energy photons. Note that since two phonons are involved in the 2D, a higher order theory is needed, and the expected resonance structure can be different, potentially having three resonances.

St/ASt measurements like these provide some opportunities for measurement science. One key application of ASt arises from the fact that many materials and/or their matrices are fluorescent. Importantly, fluorescence signals can often overwhelm or obscure St RS, but since fluorescence emission occurs at longer wavelengths than excitation, it is typically not an issue for ASt measurements; this extends the ability to generate REMs to materials and molecules that would otherwise be problematic due to fluorescence backgrounds. A second important driver of ASt measurements is that detectors are generally sensitive to a limited range of wavelengths (e.g., ≈0.4 to 1.0 μm for silicon-based detectors). Since ASt bands are blue-shifted relative to the ingoing photon-energy, their use can thus extend measurement of RS to smaller outgoing photon energies (i.e., longer wavelengths) than St RS.

Another factor that should be considered is that heating often becomes significant as laser powers increase. As an example, our use of a pulsed light source in this work leads to an instantaneous temperature greater than what would be observed for a continuous wave source of the same average power. For many measurements, it is likely important to keep the instantaneous illumination power density low enough such that the temperature is well-known and any heating derived rise is insignificant. One manner for potential compensation of a greater illumination power is to use a substrate and/or matrix with good thermal conductivity, such that generated heat is dispersed by conduction. Alternatively, since SWCNTs are so robust with temperature, it may be of practical interest to purposely heat samples so that that the ASt signal is strengthened. This could allow for the detection and identification of otherwise challenging samples, or imply greater utility of ASt measurements when high temperatures might be of interest, for example to track chemical reactions, or to track thermally driven physical changes. Of note, it is probably best in such situations to heat in an inert environment as nanotubes are less stable with temperature in the presence of oxygen. Also, while cooled samples should have sharper spectral features, ASt bands will weaken in intensity, and will become difficult to detect at low temperatures. Having St/ASt REM should provide a better assessment of temperature than fixed wavelength RS. Because REP feature breadths depend on lifetimes which are temperature dependent, it is promising to explore how REMs and REPs evolve with temperature and their correlations with the relative intensities of ASt RS bands.

In terms of our instrumentation and implications for its development, the edge filter-based setup we have used herein to block the Rayleigh scattering and transmit RS also blocks the RS close to the laser line. However, ASt bands are intrinsically strongest close to the laser line, and St bands close to the laser line such as the RB are important. So, it will be practically useful in the future to improve the performance of the instrument in regions closer to the laser line.

## Summary and Conclusions

Broadband REMs including both halves of the maps, St and ASt are obtained experimentally through a full-spectrum technique for three species of sorted SWCNT, (7,6), (7,5) and (6,5), using different sample preparation techniques. For a given band, time reversal symmetry makes St and ASt mirror images of one another up to a scaling factor. However, since ASt band intensities need pre-existing phonons, while St bands do not, the scaling factor is different for each band, the factor being activated by temperature. Elementary quantum mechanical models predict a butterfly like pattern of enhanced modes in two quadrants of the map delineated by the electronic resonance, and this pattern is indeed seen in the maps. The known asymmetric double REP peak with weaker high energy resonance is seen for the G band, but the 2D band shows a different trend; the REP structure for a general band is strong between the *E*_22_ lines, but can have a complicated structure. Laser power is a critical variable. Here, since our light source is a supercontinuum laser pulsed with a ≈10^–3^ duty cycle, it is easy to apply instantaneous power densities at which laser heating causes 100 °C temperature changes, and increases ASt band intensities, while apparently also broadening REPs. Nanotubes are robust and heating can bring the St/ASt ratio up from order 10^–3^ to order 10 for the G band, but this must be considered for comparisons of intrinsic photophysics, particularly those enabled by different sample environments. As for intensities, the *E*_22_ resonance the integrated St G band of SWCNTs is 3000× greater than the G band of HOPG. The absolute intensities of the ASt bands are determined mainly by temperature, and decrease with increasing phonon energy. Different samples showed different ASt intensities probably related mainly to the thermal conductivity of their surroundings.

## Methods

Certain equipment, instruments, software, or materials, commercial or noncommercial, are identified in this paper in order to specify the experimental procedure adequately. Such identification is not intended to imply recommendation or endorsement of any product or service by the National Institute of Standards and Technology (NIST) or the National Research Council Canada, nor is it intended to imply that the materials or equipment identified are necessarily the best available for the purpose.

### Raman Excitation Mapping

The design of the REM instrument used here is described more thoroughly in ref. [9]. Briefly, a supercontinuum white light was used as the light source (NKT Photonics SuperK Extreme EXR-15). Collimated light from the supercontinuum source was filtered to eliminate light outside the illumination band (>≈800 nm). To reject unwanted Rayleigh scattering, the beam was passed through either a short-pass edge filter (for the St part of the REM) or a long-pass filter (for the ASt part of the REM). The beam was expanded by a beam expander, and dispersed spectrally by a 300 lines per mm transmission grating. The first order diffraction was then directed through a 20× long working distance microscope objective tilted 60° from the sample normal to focus a “rainbow line” onto a flat sample. The sample was mounted on a motion control stage. The rainbow line was microns in width and mm-scale in length. A second 20× microscope objective, normal to the sample, collected the scattered light. A 45° mirror directed this light through a second edge filter, chosen to be complementary to the first edge filter. That is, if the ingoing beam used a short-pass filter, the outgoing collected beam used a complementary long-pass filter, and vice versa. For small spectral range REMs a single complementary pair of filters was used (e.g., Figure 6). Since the filter band edges are fixed this means small shifts are visible in the bottom left corner, but as the ingoing photon energy is increased, the edge moves to higher Raman shifts, blocking lower shifts. So, for the larger spectral range REMs (e.g., Figure 2) a time sequence of 8 filter pairs was used. That is, eight sets of complementary filters were used in order. This leads to a stepwise change in the cutoff when maps are combined. Here, filter changes were done by hand, which takes a few seconds. Other schemes to reject unwanted light are possible, but edge filters have near perfect transmission and excellent blocking (blocking photons at number ratios of order 10^–6^).

The filtered light was passed through a 300 lines per mm transmission grating mounted orthogonal to the first grating. The first diffraction order of this grating was focused by a tube lens onto the focal plane of a scientific cooled complementary metal-oxide semiconductor camera (Andor Neo). The python package matplotlib was used to create the plots.

### Fixed Wavelength Raman Scattering

Samples were loaded onto a motion control stage in a custom-built Raman spectroscopy microscope. A HeNe laser, which is nominally randomly polarized, 22.5 mW (Thorlabs), was cleaned up with a 633 nm laser line filter (Iridian Spectral Technologies). Power was reduced with a neutral density filter. After reflection off steering mirrors, the laser was reflected off a 633 nm notch filter (Iridian Spectral Technologies) and through a 50× long working distance microscope objective. One mW power was incident on the sample. Scattered light was collected by the same objective, passed through the notch filter, and focused by a 10 cm tube lens onto an 8 μm entrance slit on a 0.33m spectrometer (Andor Kymera 328i). A 300 lines per mm grating blazed at 500 nm was centered on the laser line and detected by a charged coupled device camera (Andor iDus 416). Exposure times were either 1, 10, or 60 s depending on the signal strength and saturation time. The data was graphed using the python matplotlib package.

### Aqueous Two-Phase Extraction Samples

Highly enriched single (*n,m*) SWCNT dispersions in 10 g/L aqueous sodium deoxycholate solutions were prepared as previously reported.^29^ Briefly, SWCNT powder (Chasm Nanotechnologies, SG65i grade lot 64 or CG400 lot PO630) was dispersed in a 20 g/L sodium deoxycholate (DOC) solution by tip sonication, purified by centrifugation and rate-zonal ultracentrifugation, concentrated into 10 g/L DOC solution by ultrafiltration, and subjected to surfactant-controlled aqueous two-polymer phase extraction (ATPE). In surfactant-controlled ATPE, the concentrations of different surfactants control the selective partition of individual SWCNT species across two spontaneously separating, water-soluble, polymer phases. After isolation of highly enriched single (*n,m*) aliquots, repeated concentration and dilution in the ultrafiltration cell (Millipore) was used to concentrate the SWCNTs, remove the ATPE polymers, and to set the DOC solution concentration to 10 g/L in H_2_O. An aliquot of the (7,6) SWCNT dispersion was further processed by filtration against a membrane (Whatman Nucleopore) followed by rinses with ethanol and water to remove surfactant; the surfactant free SWCNT film was then removed from the membrane and transferred to a silicon wafer using a published procedure^30,31^

### Conjugated Polymer Extraction Samples

The materials for conjugated polymer extraction (CPE) were: CoMoCAT SG65i purchased from Sigma-Aldrich (Cat# 773735), poly­[(9,9-dioctylfluorenyl-2,7-diyl)-*alt*-co-(6,6’-{2,2’-bipyridine})] (PFO-BPy6,6’, number-averaged molecular weight, *M*_n_ = 11.8 kDa, weight-averaged molecular weight, *M*_w_ = 39.0 kDa) purchased from American Dye Source, Inc. and Poly­(9,9-di-n-octylfluorenyl-2,7-diyl, *M*_n_ = 22.3 kDa, *M*_w_ = 53.7 kDa) (PFO), synthesized in our own laboratories. Toluene (HPLC grade) was purchased from Fisher Scientific.

The enrichment process^32,33^ for CPE was similar for both (6,5) and (7,5): for (6,5), the carbon nanotube (CNT) source (CoMoCAT SG65i, 31.2 mg) was mixed with PFO-BPy6,6’ (46.8 mg) in 25 mL of toluene. The mixture was tip-sonicated (Branson Sonifier 250) with a mini-tip of 0.48 cm (3/16 in.) at an output 30% and a duty cycle of 60% for 30 min with the container being cooled in ice water, followed by centrifugation at 1320 rad/s for 90 min (a relative centrifugal force of 18700*g*). The supernatant was filtered through Kimwipes Delicate Task Wipes to remove any loose precipitates. The enrichment was repeated for multiple cycles to maximize the yield, and the cycles were named pre, first, second, third, fourth, and so on. For (7,5), the procedure was the same except that PFO was used instead of PFO-BPy6,6’.

To make opaque, flat films for optical measurements the (6,5) and (7,5) supernatants from the third cycle for (6,5) and fourth cycle for (7,5) were filtered through PTFE membranes (pore size 0.2 μm) by gravity (without vacuum), and the filtrates were filtered one more time. The collected films were rinsed with plenty of toluene to remove excess polymers. The PTFE membranes supporting the rinsed films were taped onto a glass slide for Raman excitation mapping measurements.

## Supplementary Material



## Notes

List of acronyms used in the main text and their meanings and a supplementary figure showing the maps with the color-scale zoomed in by a factor of 10× to better show weak features (PDF)

## Abbreviations

SWCNT: single-walled carbon nanotube
RS: Raman scattering
RRS: resonant Raman scattering
PL: photoluminescence
PLE: photoluminescence excitation
REP: resonant excitation profile
REM: Raman excitation map
RB: radial breathing
St: stokes
ASt: anti-stokes
FS-REM: full spectrum Raman excitation mapping
ATPE: aqueous two-phase extraction
CPE: conjugated polymer extraction
eV: electron volt
Ry: Rayleigh
HeNe: helium neon
OD: optical density
HOPG: highly ordered pyrolytic graphite
