# Supplementary material for: The Resonance Structure of Raman Scattering for Emitted and Absorbed Phonons in Chirality-Pure Carbon Nanotube Films
Source: ACS Nano. 2025 Nov 14;19(46):39793–804. doi: 10.1021/acsnano.5c12607 (PMC12659417; doi:10.1021/acsnano.5c12607)
Supplement: Supplementary file 1 [file nn5c12607_si_001.pdf]

Supporting Information for:

The Resonance Structure of Raman Scattering for Emitted and Absorbed Phonons in Chirality-Pure Carbon Nanotube Films

Paul Finnie,†, Adam Wind†,¥, Jianying Ouyang†, Pavel Shapturenka §, Jeffrey A. Fagan§*

†Quantum and Nanotechnologies Research Centre, National Research Council Canada, 1200 Montreal Road, Ottawa, Ontario, K1A 0R6, Canada

¥University of Waterloo, 200 University Avenue West, Waterloo, ON, N2L 3G1, Canada

§Materials Science and Engineering Division, National Institute of Standards and Technology (NIST), Gaithersburg, MD 20899, USA

Certain commercial equipment, instruments or materials are identified in this paper in order to adequately specify experimental details. Such identification does not imply recommendation or endorsement by National Institute of Standards and Technology (NIST) or by the National Research Council Canada (NRC), nor does it imply that the materials or equipment are necessarily the best available for the purpose.

Table of Contents:

1. Acronyms
2. Scaled Raman excitation maps

1. Acronyms:

SWCNT, single-walled carbon nanotube;
RS, Raman scattering;
RRS, Resonant Raman Scattering;
PL, photoluminescence;
PLE, photoluminescence excitation;
REP, resonant excitation profile;

REM, Raman excitation map;
RB, radial breathing; St, Stokes;
ASt, Anti-Stokes;
FS-REM, Full spectrum Raman excitation mapping;
ATPE, aqueous two-phase extraction;
CPE, conjugated polymer extraction;
eV, electron Volt;
Ry, Rayleigh;
HeNe, Helium Neon;
cw, continuous wave;
OD, optical density;
HOPG, highly ordered pyrolytic graphite

2. Scaled Raman excitation maps

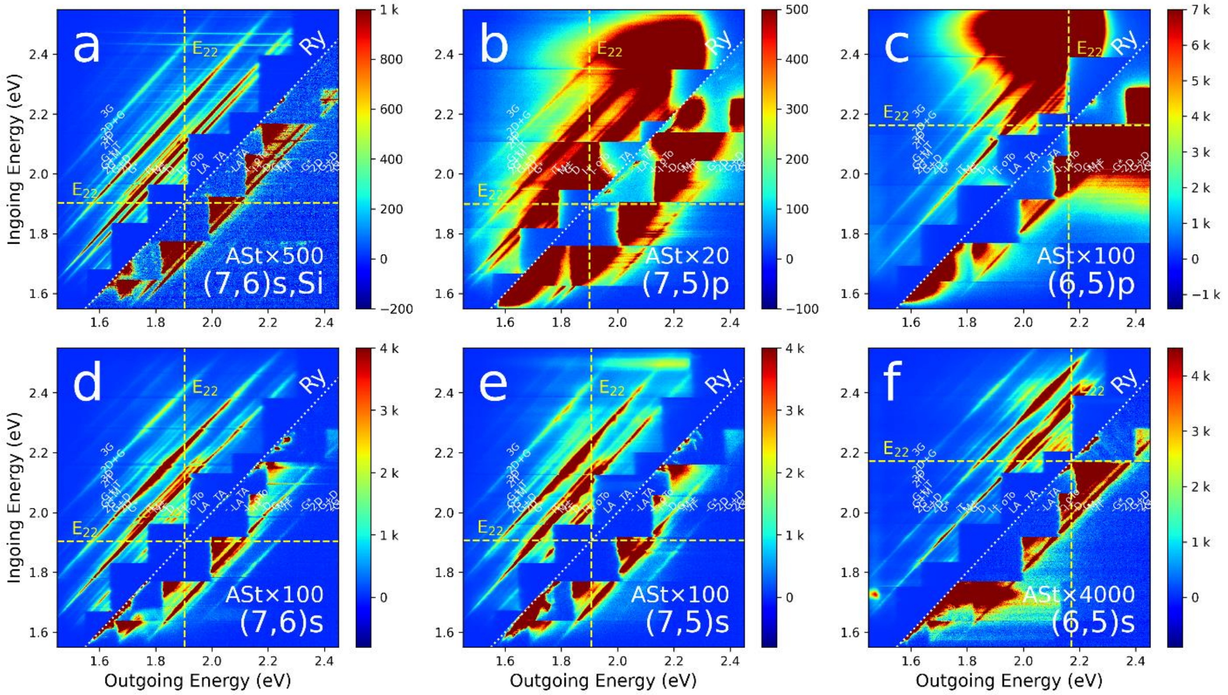

Figure S1 Saturated Experimental Stokes/Anti-Stokes Raman Excitation Maps.

The experimental Raman Excitation Maps of Figure 2 are shown with the color-scale zoomed in by a factor of 10×. Stronger features are saturated on this scale but faint features are more visible. Otherwise, all plots are the same and labelled in the same way as Figure 2.
